# Extending Thrombolysis in Acute Ischemic Stroke to Primary Care: Early Experiences with a Network-Based Teleneurology Approach

**DOI:** 10.3390/neurolint14010012

**Published:** 2022-01-21

**Authors:** Francesco Corea, Monica Acciarresi, Laura Bernetti, Pierluigi Brustenghi, Arianna Guidubaldi, Mariangela Maiotti, Sara Micheli, Vilma Pierini, Alessio Gamboni, Giuseppe Calabrò, Chiara Busti, Cesare Magistrato, Gianluca Proietti-Silvestri, Massimo Bracaccia, Valeria Caso, Mauro Zampolini

**Affiliations:** 1 Stroke Unit, San Giovanni Battista Hospital, 06034 Foligno, Italy; monica.acciarresi@uslumbria2.it (M.A.); laura.bernetti@uslumbria2.it (L.B.); pierluigi.brustenghi@uslumbria2.it (P.B.); arianna.guidubaldi@uslumbria2.it (A.G.); mariangela.maiotti@uslumbria2.it (M.M.); sara.micheli@uslumbria2.it (S.M.); vilma.pierini@uslumbria2.it (V.P.); mauro.zampolini@uslumbria2.it (M.Z.); 2 Emergency Department, San Giovanni Battista Hospital, 06034 Foligno, Italy; alessio.gamboni@uslumbria2.it (A.G.); giuseppe.calabro@uslumbria2.it (G.C.); chiara.busti@uslumbria2.it (C.B.); 3 Emergency Department, Santa Maria della Stella Hospital, 05018 Orvieto, Italy; cesare.magistrato@uslumbria2.it; 4 Emergency Department, San Matteo degli Infermi Hospital, 06049 Spoleto, Italy; gianluca.proiettisilvestri@uslumbria2.it; 5 Emergency Department and Internal Medicine, Santa Maria della Stella Hospital, 05018 Orvieto, Italy; massimo.bracaccia@uslumbria2.it; 6 Stroke Unit, Santa Maria Misericordia Hospital, 06129 Perugia, Italy; valeria.caso@ospedale.perugia.it

**Keywords:** teleneurology, stroke, telemedicine, thrombolysis

## Abstract

Background and Purpose—Systemic thrombolysis represents the main proven therapy for acute ischemic stroke, but safe treatment is reported only in well-established stroke units. To extend the use of tissue plasminogen activator (tPA) treatment in primary care hospitals on isolated areas through telemedic was the purpose of specific initiatives in southern Umbria, Italy. Methods—The stroke center of Foligno established a telestroke network to provide consultations for three local hospitals in southern Umbria. The telemedic system consists of a digital network that includes a two-way video conference system and imaging sharing. The main network hospital established specialized stroke wards/teams in which qualified teams treat acute stroke patients. Physicians in these hospitals are able to contact the stroke centers 24 h per day. Quality data are available to support the safe implementation of the stroke procedures. Those available from governmental authorities and local datasets are volume of hospitalization, in-hospital mortality, 30-days mortality, and discharge setting. Objective of the study was to assess the annual hospitalization volume in both the hub and spoke hospitals for ischemic stroke and appraise the performance of the network after the introduction of the telestroke system. Results—A total of 225 systemic thrombolyses were performed in time period indicated above all hospitals. In the main spoke hospital, 41 procedures were performed after teleconsultations were made available. The thrombolysis rate in the hub hospital ranged between 10% in 2016 and 20% in 2019, while in the spoke hospital was below 5% in 2016 and raised to 15% in 2019. The statistically significant difference, in the number of procedures, between hub and spoke in the beginning of the observation time disappeared after introduction of the telestroke network. No increase of the mortality was found. Conclusions—The present data suggest that systemic thrombolysis indicated via stroke experts in the setting of teleconsultation shows similar complication rates to those reported in the National Institute of Neurological Disorders and Stroke trial. Therefore, tPA treatment is also safe in this context and can be extended to primary hospitals.

## 1. Introduction

Telemedicine is increasingly used for acute stroke care, making neurological expertise available in primary hospitals [1].

Although included in continental recommendations there are a few real-life data about telemedicine’s performance on thrombolysis and outcome measures at treating hospitals. We aimed to assess the performance of a stroke network with telemedical support on care, according to acute phase processes and short-term outcome [2,3,4].

In Umbria, an Italian region in central Italy, about one-third of the population lives in rural or internal areas with reduced access to time-dependent therapy for stroke care.

The territorial area of the local health care provider Usl Umbria 2 includes 54 municipalities with a total area of 4152 km^2^ and with an assisted population of 382,575 inhabitants. In the competence area 7 hospitals operate, and only one of these, San Giovanni Battista Hospital in Foligno, has a fully equipped emergency department with a stroke pathway for both thrombolysis and selection to thrombectomy, which is provided elsewhere. Among the other hospitals, the more relevant for position and volume of cases and without a full stroke center is the Santa Maria della Stella hospital in Orvieto (municipality with 20,000 inhabitants, stroke hospitalization volume around 100/year). This primary hospital in the border with Lazio is suffering a poor road network connection to the rest of the county.

In general, according to data available by the Italian neurology society, the ratio between stroke units and inhabitants in Umbria is adequate and equal to 112% coverage (ideal ratio: one stroke unit per 200,000). Other Italian Regions, such as Campania, have a ratio of around 10%. However, in Umbria the number of recorded thrombolysis treatments on all ischemic strokes, as observed by the European Register of the Karolinska Institute, is half the theoretical. As thought, only half of the ischemic stroke patients had access to the acute treatment. Other Italian Regions, such as Veneto, reached a more satisfactory result (almost the 80% of procedures) on potential annual cases [5,6].

For this reason, during the past years in Umbria, many initiatives have been undertaken to enlarge the accessibility to stroke care as well as to bridge the infrastructural gap.

Since 2014, in USL Umbria two catchment areas, stroke has been “coded” as an independent therapeutic diagnostic pathway and a reorganization of the emergency department pathway has been carried out. The ability to centralize acute stroke patients was guaranteed through facilitated patients dispatchment by spoke centers to Foligno ensuring the presence of trained pre-screening with emergency medical and paramedic staff in each spoke hospital, meanwhile, also adopting the policy of “always available stroke beds” at the hub hospital of Foligno.

To optimize the stroke pathway also the three primary hospitals, Orvieto, Norcia, and Spoleto without a fully specialized stroke care were included in a network with telemedical support by Foligno general hospital. Stroke patients admitted consecutively to one of the participating hospitals between January 2017 and 31 March 2021, were included in the study. Patients were assessed in the same manner and were followed up for 30-days mortality and discharge setting. Outcome was defined by death, institutional care, or severe disability (admission in a rehab facility or nursing home). Predefined indicators for quality of acute stroke care were achieved [7,8].

**Figure 1:**
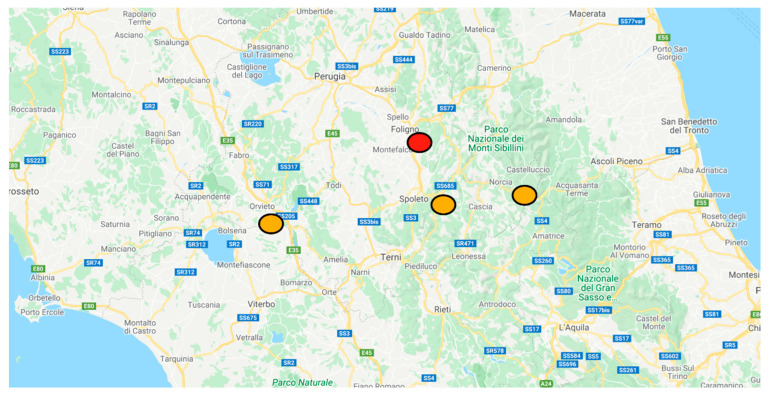
Geographical catchment area, and location of hospitals in USL Umbria 2 area, Adapted and modified from google maps. In red the hub hospital of Foligno, in orange to the left the spoke of Orvieto, in the middle Spoleto and to the right Norcia. https://www.google.it/maps/search/ospedali/@42.9888504,12.6977109,10z/data=!3m1!4b1 last accessed on 5 December 2021.

Objective of the study was to assess the annual hospitalizations volume in both the hub and spoke hospitals for ischemic stroke and appraise the annual thrombolysis rate after the introduction of the telestroke system (map of hospitals on Figure 1). The secondary objective was to assess the performance of the network using the 30-day mortality rate; this is to exclude any potential harm on short-term vital outcome.

## 2. Methods

### 2.1. Data

The annual hospitalization volume for ischemic stroke in each hospital was extracted from the Progetto Nazionale Esiti (PNE) database of the Italian Health Service (SSN) offering open access for disease-specific data from all health care providers [9] (http://www.agenas.it/ Last accessed on 5 December 2021). The larger time window available was from 2012 to 2019. Data are processed by the system 18 years after the closure of the year. Hospitals with a volume of stroke below 20 cases per year were excluded from the national quality database considering them as residual.

There were retrospectively extracted data in the time interval 2016–2021 (first quarter 2021) on annual volume of stroke patients admitted, cumulative number of thrombolytic treatment, in hospital overall mortality and discharge setting. The annual number of thrombolysis and discharge setting, for both hub and spoke hospitals, were obtained using the informatics system of the health care provider looking for DRG coded as 99.10—Injection or infusion of thrombolytic agent (ICD-9-CM Vol. 3 Procedure Codes).

### 2.2. Teleneurology System

Within the project for Stroke care support in USL Umbria 2 a telemedical system was progressively introduced since 2017 in three primary care hospitals. An ED triage algorithm was established for all patients with acute stroke. In addition to these measures, from January 2017, a teleconsultation connection between Orvieto Emergency Department and Foligno Neurology was introduced with a medical video-conference system (Meytec TM, Werneuchen, Germany) for patients’ real-time assessment.

Additionally, during the earthquake emergency in central Italy, a similar system was deployed in the hospital of Norcia 2018 and later in 2019 also the hospital of Spoleto. In these sites the use of system was only adopted for inter hospital dispatchment.

The stroke care pathway helps the emergency physicians of any spoke to activate the neurologist on duty, when available in house (usually 6 h coverage during the day), or the on-call neurologist during night/holiday, in the hub hospital of Foligno.

The neurologist, briefly discussing the case on the phone, agrees to perform the teleconsultation with:

- Access to Orvieto CT images via RIS/PACS system.
- Patient televisit in the presence of medical and/or nursing staff in Orvieto Emergency Department.

After discussing the clinical case and interviewing, if needed, the patient, family members, emergency transport paramedics for other details, the neurologist provides a written report to the spoke hospital offering diagnostic and best therapeutical options.

### 2.3. Statistics and Data Management

We applied descriptive statistics. Due to non-normal distribution of cases, data are presented as median and interquartile range. A two-sided Fisher’s exact test was performed to confront numerosity of patients undergoing thrombolysis in the hub and spoke hospital each year. A *p*-value < 0.05 was considered significant. All patient data are derived from our anonymized and project-related quality register. According to legislation, no patient consent is required for documentation in routine observational quality registries.

## 3. Results

### 3.1. Volume of Admissions

**Figure 2:**
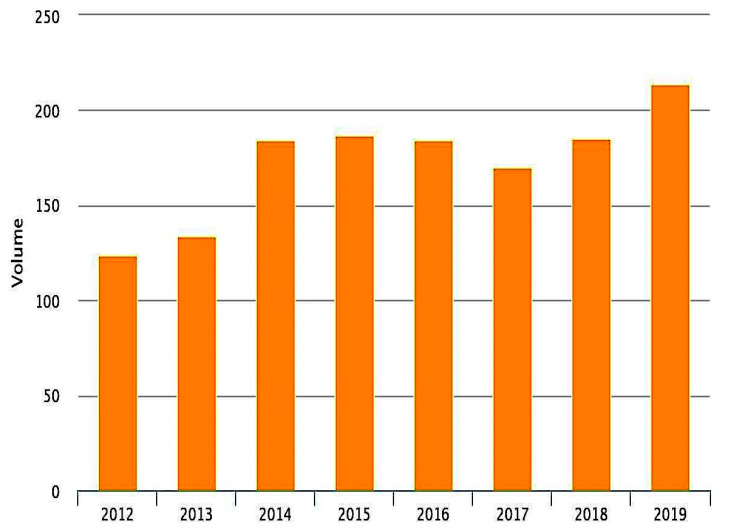
Number of acute ischemic stroke admitted in the hub hospital of San Giovanni Battista Hospital in Foligno from 2012 to 2019.

**Figure 3:**
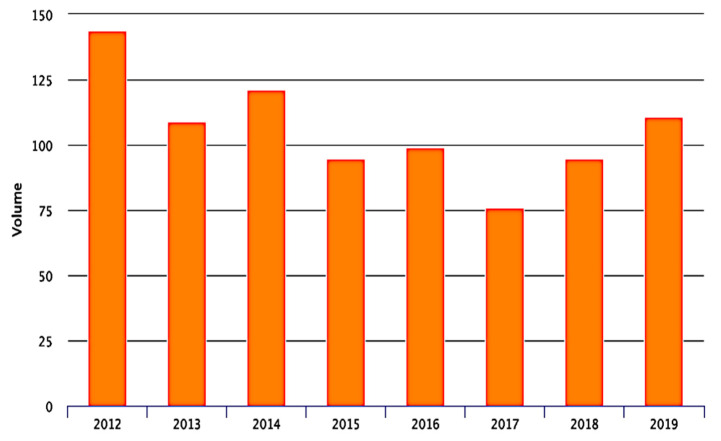
Number of acute ischemic stroke admitted in the spoke hospital of Santa Maria della Stella Hospital in Orvieto from 2012 to 2019.

The volume of admission during the observation time according to PNE project is shown in Figure 2 and Figure 3. The annual ischemic stroke admission volume of the hub hospital ranges from 184 cases in 2016 to 214 in 2019 while in the main spoke volume ranged between 76 cases in 2016 and 111 in 2019. The number of stroke patients admitted in other minor hospital was residual (below 20 cases) and thereby was excluded from the national data quality analysis.

**Figure 4:**
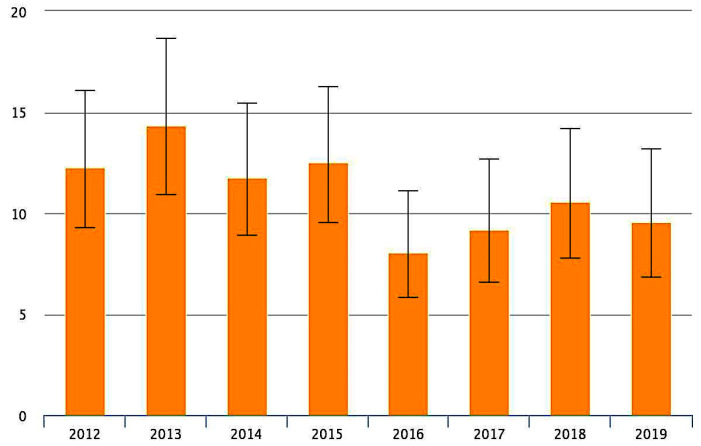
Overall, 30-days mortality rate in the county for patients suffering for an ischemic stroke from 2012 to 2019. Output included IC 95%.

The overall 30-days mortality according to PNE system for ischemic stroke in the county is reported in Figure 4. We can observe a peak of mortality in 2013 around 14% and later the percentage drops to below 10%.

### 3.2. Thrombolytic Procedures

**Figure 5:**
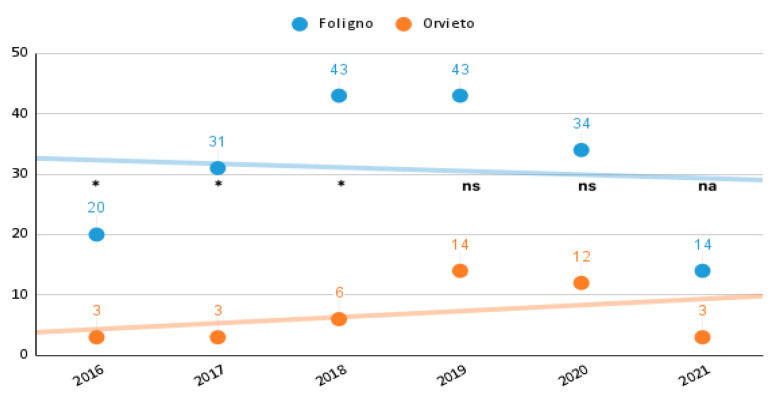
Number of acute ischemic stroke treated with tPA in the participating hospitals from 2016 to the first quarter 2021. * two-sided Fisher’s exact test with *p* value <0.05; ns: two-sided Fisher’s exact test non-significant *p* value; na: not applicable.

There were 225 cases treated with thrombolysis, in the time frame investigated, admitted in the hospitals of the network. The annual number of procedures is reported in Figure 5. The hub hospital performed the majority of procedures, 185, while the spoke center treated 41 cases.

Baseline characteristics, risk factors, and symptoms of the 225 cases are given in Table 1. We observed a statistically significant difference in the number of thrombolysis performed in 2016 (23 cases), respectively, 20 in the hub 3 in the spoke hospital *p* < 0.05); in 2017 (34 cases) respectively 31 in the hub 3 in the spoke (*p* < 0.05); 49 in 2018 respectively 43 and 6 (*p* < 0.05). There was no significant difference in later years in the number of thrombolysis performed: 57 in 2019 respectively 43 and 14 (*p* value = ns); 46 patients were treated in 2020, respectively, 34 and 12 (*p* value = ns), in the first quarter on 2021 17 treated patients, respectively, 14 in the hub and 3 in the spoke (not applicable due to partial data).

The thrombolysis rate in the hub hospital ranged between 10% in 2016 and 20% in 2019, while in the spoke hospital was below 5% in 2016 and raised to 15% in 2019.

The 30-days mortality rate for ischemic stroke in the database for hospitalized patients ranged between 8.06% in 2016 and 9.54% in 2019.

### 3.3. Distribution of Hospital Discharge

**Figure 6:**
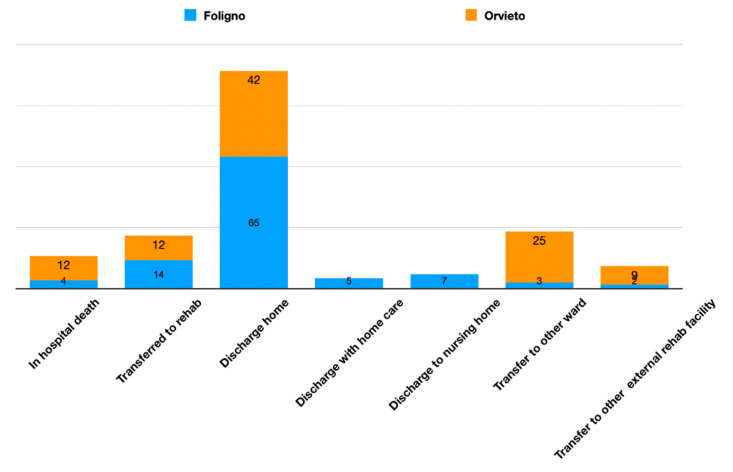
Patients suffering an ischemic stroke treated with tPA and discharge setting. Relative percentages for each hospital.

Above 225 subjects undergoing thrombolysis for ischemic stroke in the active stroke units (Foligno as hub stroke unit and Orvieto as spoke Telestroke unit) a home discharge was reported in 17 cases (41.4%) in the spoke and 121 (65%) in the hub while in-hospital death was observed in 5 cases (12.1%) in the spoke and 12 (4.3%) in the hub; subjects transfer to an external rehab facility was reported in 5 cases (12.1%) in the spoke and 27 cases (14.9%) in the hub; discharge with home care was reported 5 cases (2.7%) while no cases in the spoke; discharge to nursery home was reported in 13 cases (7%) in the hub no cases in the spoke; transfer to another hospital ward was reported in 10 cases (24%) in the spoke and in 7 cases (3.7%) in the hub; the transfer to a internal rehab facility was reported in 4 cases (9.7%) in the spoke hospital and in 4 cases (2.1%) in the hub hospital (Figure 6).

## 4. Discussion

Our quality data analysis confirm the current observation of a positive effect of the Teleneurology program on supporting the decision-making process of thrombolysis in Southern Umbria. Moreover, a benefit in volumes of admissions was observed.

In the hub hospital of Foligno the volume of admission doubled in the analysis period. A larger number of patients skipped minor hospitals being treated in the hub.

The main spoke hospital of Orvieto demonstrated a drop of volume until 2017 with a minimum of 75 cases admitted. After introduction of the Teleneurology support the number of cases admitted increased to above 100 cases per year. Higher confidence of the medical staff avoided futile transfers. This is the first analysis of safety of a Teleneurology system on substantial number of thrombolyses in stroke conducted after telemedic evaluation in Italy and southern continental Europe [10,11,12,13]. However, only small samples of telethrombolyses have been reported elsewhere [13,14,15].

The present analysis demonstrates that systemic tPA thrombolysis can be administered safely in primary hospitals if indicated via teleconsultation by experienced stroke neurologists and emergency medicine medical personnel.

According to quality system data, the 30-days mortality rate in the spoke hospital was higher (12%) (Figure S1) but remained comparable to the natural history of ischemic stroke reported for hospitals experienced in thrombolysis [16].

The percentage of patients receiving tPA treatment in the first 12 months of the ongoing project was still lower than the rates reported in established stroke units, but the increasing numbers in the later 3 years indicate a clear improvement. This improvement is related to better prehospital stroke management achieved by the medical staff of the primary hospital.

Effective “telethrombolysis” in stroke requires a 24-h, on-demand teleconsultation service equipped with trained stroke experts. In our project, the expenses for purchasing the telemedicine system amounted to 180,000 €. Given the calculated savings of between $3800 and $5000 (3200 € to 4200 €) per thrombolysis, the absolute increase of 35 tPA treatments in the spoke hospital within 2 year would produce a reduction of subsequent costs between 133,000 € and 147,000 €. The medical staff of the hub stroke unit provides consultation during on-call duties. In case larger volumes of tele consultations are reached, a specific budgetary program for dedicated personnel may be needed.

Other strategies such as implementation of helicopter transport systems are more expensive, and in case of delayed hospital admission the time window may be too narrow to guarantee the delivery of time-dependent therapy [17,18,19].

There are several limitations to our study. First, the clinical data of the population are retrospective and very limited (e.g., missing on any radiological finding). Second, only ten years were taken into consideration and were influenced by the COVID-19 pandemic in the last months. Strengths include that all data concerning hospitalization volumes, vital outcome were extracted by an independent health care authority.

It would have been interesting to explore other performance markers such as: onset-to-needle time or door-to-needle times in each hospital. In our study we opted to focus only on the death rate and discharge setting. Our data quality analysis offers highly solid data in terms of 30-day mortality and volume of admissions extracted from an independent governmental agency [9]. No data are available on timing of stroke admissions and therapy. A serious increase of mortality rates or abnormal rates of discharge in nursing home may be enough to give rise to major safety concerns on the Telestroke network performance.

Although national political authorities pushed toward a telemedicine revolution, many barriers still stand. The availability of digital health-integrated standard operating procedures (SOPs) for main clinical lines would support the decision-making process and minimize errors. Medical educational models should be reorganized on many levels, and stakeholders should reserve an open-minded approach to this process, in order to avoid the risk of being overwhelmed or miss useful opportunities [20].

Moreover, COVID-19 may have effects on the study in the last 12 months. On 29 February 2020, the Italian Minister of Health issued national guidelines on how to address the COVID-19 emergency. Stroke management was affected and required changes, basically resulting in the need to prioritize the ongoing COVID-19 emergency. In the most affected regions, the closure of departments and hospitals led to a complete reorganization of previously functioning stroke networks. With the closure of several stroke units and stroke centers, the transportation time to hospital lengthened significantly, especially for the outlying populations. The mentioned effects showed mild consequences in central Italy and in Umbria specifically, also the rate of thrombolysis was slightly reduced [21].

We expected initially a further positive effect toward telemedicine use during the COVID-19 pandemic. Unexpectedly in our case the major waves of COVID-19 and the subsequent re-organization of the spoke hospital led to a reduction of inter-hospital teleconsults. While we observed an increase of intra-hospital telemedicine in the hub between COVID grey areas and standard neurology ward.

The present data suggest that systemic thrombolysis indicated via stroke experts in a teleconsultation setting shows similar complication rates to those reported in the clinical trials National Institute of Neurological Disorders and Stroke trial. Therefore, tPA treatment is also safe in this context and can be extended to nonurban areas [22].

## Figures and Tables

**Table 1:** Patients characteristics on acute ischemic stroke admitted in the participating hospitals and treated with.v. tPA treatments from 2016 to first quarter 2021.

	n	N	%
Age (median; IQR [years])	67 (54–79)		
Female sex	121	225	54%
Risk factors			
arterial hypertension	130	225	58%
diabetes mellitus	45	225	20%
hypercholesterinemia	78	225	35%
(ex-)smoker	31	225	14%
atrial fibrilation	33	225	14%

IQR indicates interquartile range.

## Data Availability

Dataset for 30-days mortality and volume of admission are available at governmental web site https://pne.agenas.it Last accessed on 5 December 2021.

## Supplementary Materials

The following supporting information can be downloaded at: https://www.mdpi.com/article/10.3390/neurolint14010012/s1, Figure S1: Charts on 30-day mortality of each hub/spokes hospital.
