# Supplementary figures and images for: Extending Thrombolysis in Acute Ischemic Stroke to Primary Care: Early Experiences with a Network-Based Teleneurology Approach
Source: Neurol Int. 2022 Jan 21;14(1):164–73. doi: 10.3390/neurolint14010012 (PMC8884013; doi:10.3390/neurolint14010012)

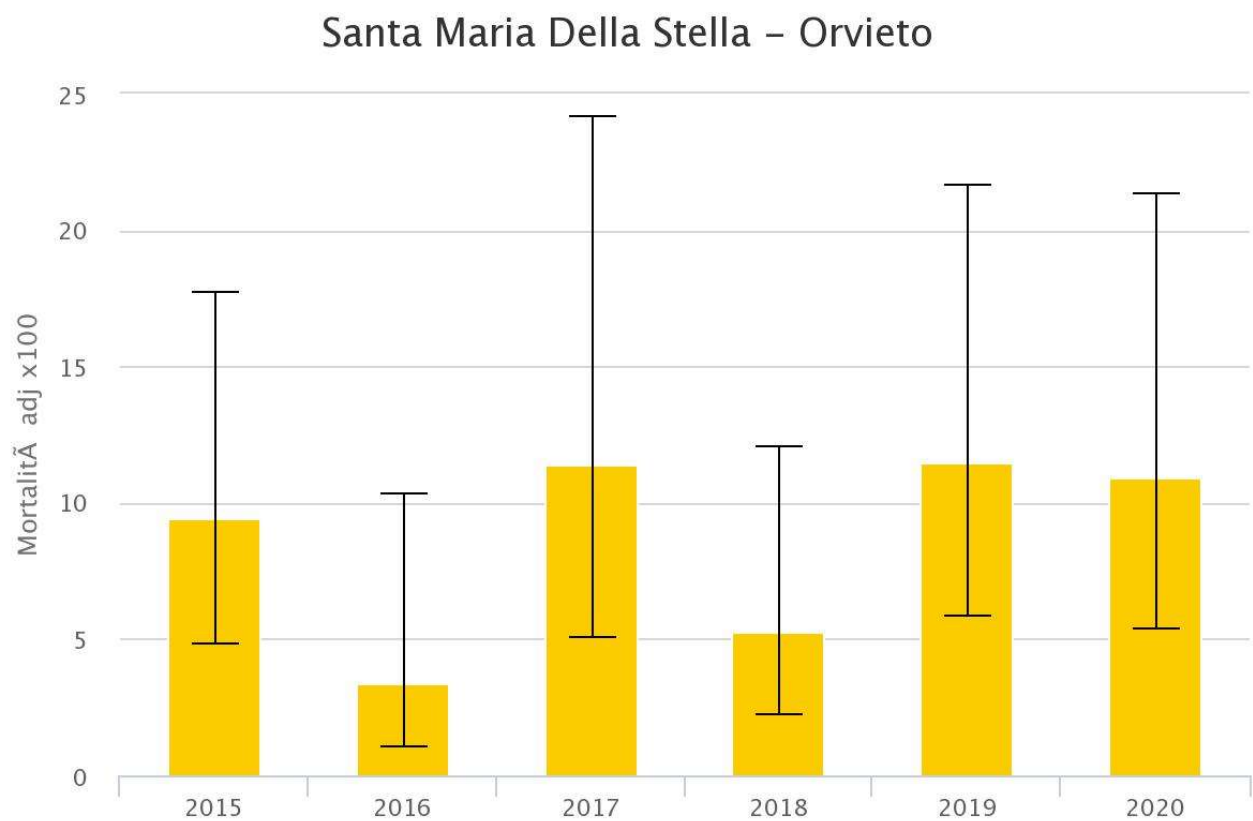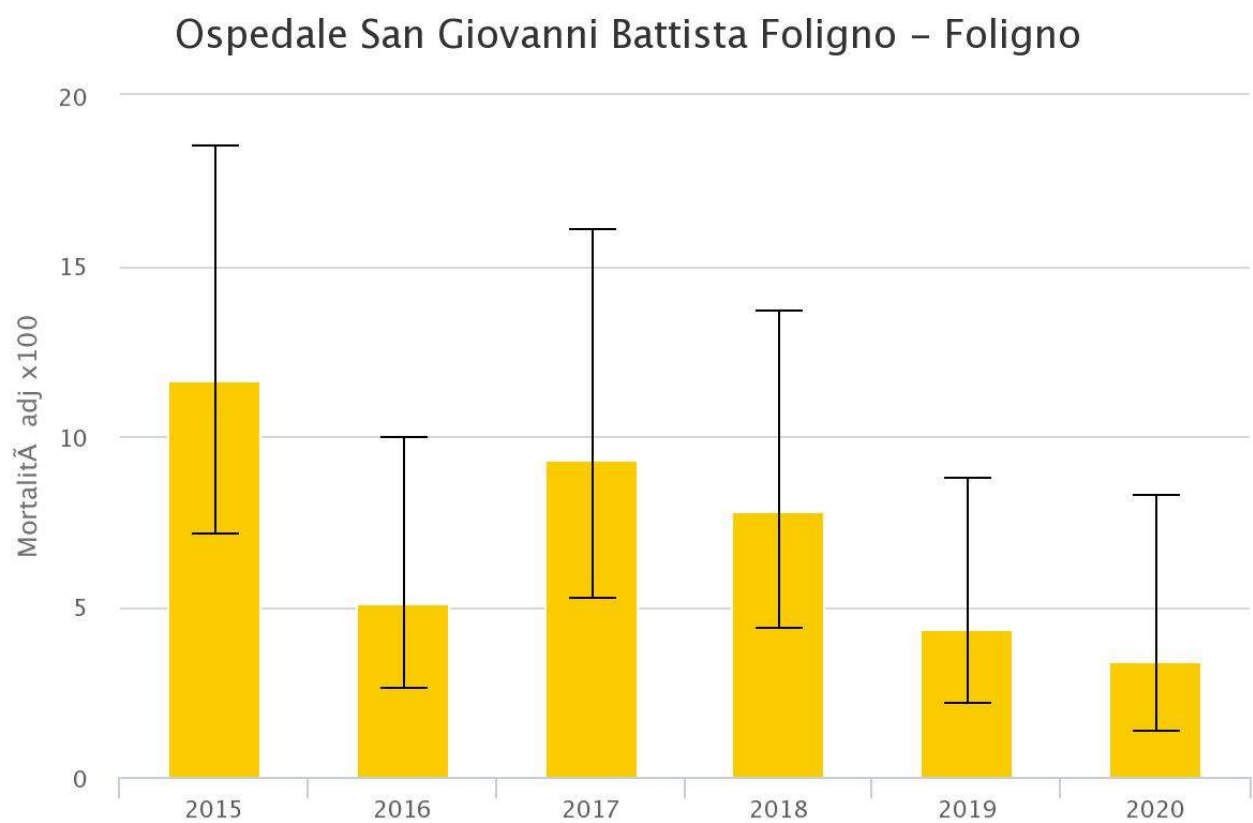

**Figure S1.** Two charts on 30-day mortality trends in each of the participating hospital.

Supplement: Supplementary file 1 [file neurolint-14-00012-s001.zip › neurolint-1514109-supplementary.pdf]
